# From mechanosensing to immune regulation: mechanisms of acupuncture signal initiation and amplification mediated by macrophages

**DOI:** 10.3389/fimmu.2026.1744045

**Published:** 2026-02-16

**Authors:** Wenru Sheng, Rui Wang, Xiqing Xue, Pan Zhao, Jingwen Zhang, Yiider Tseng

**Affiliations:** 1 Innovative Institute of Chinese Medicine and Pharmaceutical Science, Shandong University of Traditional Chinese Medicine, Jinan, Shandong, China; 2 College of Traditional Chinese Medicine, Shandong University of Traditional Chinese Medicine, Jinan, Shandong, China; 3 College of Acupuncture and Tuina, Shandong University of Traditional Chinese Medicine, Jinan, Shandong, China; 4 Institute of Acupuncture and Moxibustion, Shandong University of Traditional Chinese Medicine, Jinan, Shandong, China

**Keywords:** acupuncture initiation, immune regulation, macrophages, mechanotransduction, signal amplification

## Abstract

The core mechanism of acupuncture therapy lies in converting local mechanical stimulation into systemic physiological regulatory effects. Building on this concept, this review highlights the central role of macrophages in this mechanotransduction event, mainly occurring in the acupoint(s). During acupuncture, the practitioner’s manipulation techniques, such as lifting-thrusting and twisting, cause significant mechanical stress at an acupoint through the entanglement and traction of collagen fibers through the acupuncture needle. This physical signal is transmitted through the extracellular matrix (ECM) and delivered to the mechanosensitive cells, such as fibroblasts and macrophages. Concurrently, while fibroblasts receive the mechanical stimuli, they also release alarmin proteins, such as interleukin-33 (IL-33), to further regulate macrophages’ activities. As a key mechanical sensing and effect unit, macrophages perceive mechanical signals through multiple pathways, including Piezo1, transient receptor potential vanilloid 4 (TRPV4) mechanically sensitive channels, the integrin family of mechanotransduction receptors, and podosomes on the cell body. These pathways promptly initiate intracellular Ca^2^ fluctuations and promote the Yes−associated protein (YAP) and transcriptional co−activator with PDZ−binding motif (TAZ) for their nuclear translocation, as well as induce other mechanisms, generating a cascade reaction to activate macrophages. After activation, macrophages effectively recruit neutrophils and monocytes by coordinating the chemokine network and dominate the resolution of inflammation and the initiation of tissue repair via dynamic polarization between M1 and M2 phenotypes. Additionally, they regulate T cell-mediated adaptive immune responses through antigen presentation and other means, and collaborate with fibroblasts to promote the remodeling and repair of the ECM. This article focuses on providing a systematic perspective on the cellular and molecular basis of acupuncture initiation through the response of macrophages to acupuncture signals and their regulation of the immune network.

## 1 Introduction

Acupuncture is a treasure of Chinese medicine. With a history of application spanning thousands of years, it has been recognized in 196 countries and regions around the world for its remarkable therapeutic effects and has become one of the most influential forms of traditional medical therapies (1). In 2010, UNESCO also included acupuncture in the Representative List of the Intangible Cultural Heritage of Humanity (2). Currently, acupuncture in clinical practice mainly falls into two types: manual acupuncture and electroacupuncture. Although both types rely on needle insertion into the acupoint, they differ significantly in their mechanisms of action, operational modes, and clinical application (3, 4). Manual acupuncture, the mainstream form of traditional acupuncture therapy, depends on the operator’s manual techniques to exert therapeutic effects. Its core mechanism lies in applying direct, fine mechanical stimulation to the subcutaneous tissue of the acupoint area through techniques such as twisting and lifting the filiform needle, thereby altering the structure and physical properties of the local ECM. These mechanical signals can further be transmitted to the cells attaching on the ECM, thereby regulating those cells’ arrangement, morphology, and functional state, and giving rise to the changes in the microenvironment. Ultimately, these effects lead to the modulation of immune regulation and neural conduction. On the other hand, electroacupuncture, a modern acupuncture method derived from manual acupuncture, enhances the stimulation to the subcutaneous tissue of the acupoint area by connecting the needle handle to an electrode, hence applying controllable current to the acupoint, affecting nerve fibers, muscle tissues, and other electrically sensitive cells. The application of current alters the membrane potentials of neuron cells and activates the voltage-gated ion channels on their membranes, thereby interfering with the conduction and integration of nerve impulses. Although both methods have their unique features, the focus of this study lies in understanding the initiation mechanism of hand acupuncture therapy.

A large amount of clinical evidence in various diseases has affirmed the therapeutic effect of acupuncture; the modern biological mechanism of the sustained immunomodulatory effect after needle withdrawal remains to be systematically clarified. In recent years, progress in cell mechanics has provided a new perspective for understanding the effects of acupuncture. The mechanical stresses involved in acupuncture manipulation, such as lifting-thrusting and twisting, can cause local tissue deformation at the acupoint, activate the resident cells in the ECM, and induce a series of biochemical responses. However, which cells sense mechanical signals at the early stage of acupuncture, participate in initiating the immune cascade reaction, and mediate immune regulation remain key scientific questions.

**Figure 1:**
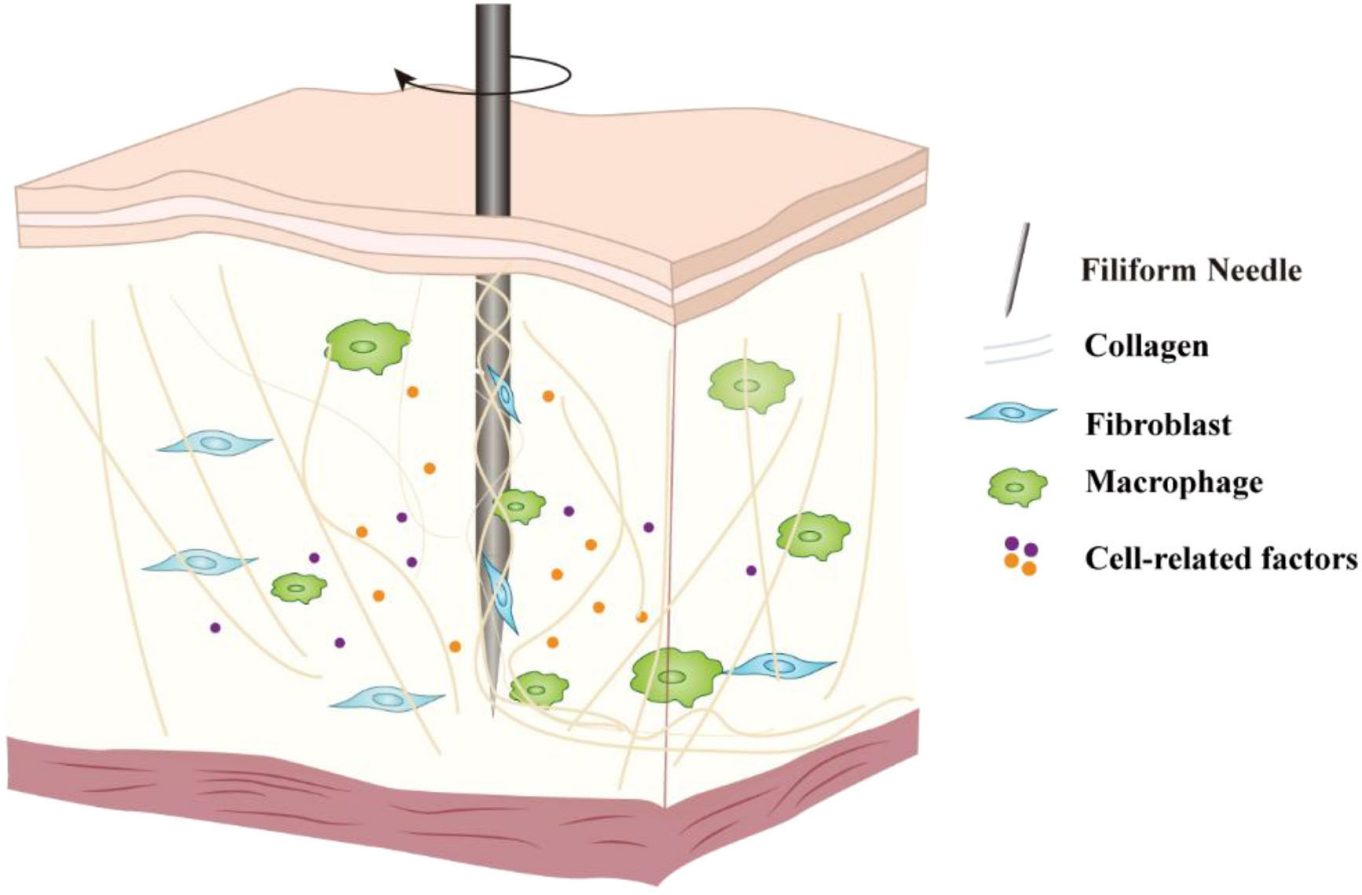
Schematic diagram of the cellular response initiated by acupuncture. The mechanical signals generated by the lifting-thrusting and twisting operations of the needle cause changes in the extracellular matrix structure at the acupoint, which are then perceived by core mechanosensitive cells such as fibroblasts and macrophages, triggering intracellular signal transduction and the secretion of biological factors, thereby amplifying the acupuncture signal.

Based on the current understanding of cell mechanics, fibroblasts and macrophages may be the first line of cells change in their spatial distribution and dynamics in the complex cellular network within the acupoint area to directly respond to the mechanical stimulation of acupuncture (**Figure 1**). Fibroblasts, as the main mechanical sensing units in connective tissue, undergo deformation and displacement when the needle wraps around and pulls on collagen fibers, directly receiving mechanical stimulation, initiating dynamic signal transduction, and triggering early biological responses. Macrophages, as resident immune cells in the acupoint area, also sense mechanical stress in the early stage of needling, participating in the rapid transduction of signals and the initiation of immune responses.

This review focuses on discussing the dynamic role of macrophages in the process of initiating acupuncture signals, systematically sorting out how they perceive acupuncture stimulation through mechanically sensitive channels, how they collaborate with fibroblasts and other cells to construct an immune microenvironment in the acupoint area, and how they guide the recruitment and functional polarization of subsequent immune cells. The aim is to reveal the dynamic role of macrophages in the acupuncture effect, explain the biological basis of acupunctural immune regulation, and provide theoretical support for the occurrence of long-term clinical efficacy.

## 2 Acupuncture mechanics initiates cellular responses through ECM mediation

### 2.1 Foundation of acupoint structure and mechanical transmission

As the key action sites for acupuncture treatment, the structural characteristics of acupoint tissues provide the morphological basis for the initiation and transduction of acupuncture signals. Acupoints are three-dimensional functional units mainly composed of multiple layers of connective tissues, including the epidermis, dermis, subcutaneous loose connective tissue layer, and fascial network layer, with the muscle layer beneath. Additionally, complex tissue structures, such as nerve and vascular bundles that traverse and distribute within acupoints (4). Among them, connective tissue plays a crucial role in the mechanical signal pathway and response (5). The dermis is composed of dense connective tissue, rich in cells involved in immune mechanisms such as fibroblasts, mast cells, macrophages, and dendritic cells (6). Furthermore, it is distributed with multiple neural cell-riched mechanorecepto (such as free nerve endings, Pacinian corpuscles, and Meissner’s corpuscles), constituting the primary stratification for mechanical stimulus perception. Meanwhile, the subcutaneous loose connective tissue layer and fascial network layer provide a structural basis for the transmission of mechanical signals. The matrix components within them, such as collagen fibers, elastic fibers, and hyaluronic acid, together form a microenvironment rich in mechanical properties, directly influencing cell behavior and signal propagation.

### 2.2 Extracellular matrix-mediated mechanical signal transduction

The ECM is a three-dimensional dynamic network constructed by collagen fibers composed of collagen, elastin, glycosaminoglycans, proteoglycans, and glycoproteins such as fibronectin and laminin (7). Besides providing structural support and elasticity for mesenchymal tissues, it also participates in the regulation of tissue homeostasis through continuous remodeling (8). During acupuncture, the needle entangles with collagen fibers, resulting in mechanical coupling, causing tissue deformation and tension changes, thereby altering the mechanical microenvironment of the ECM. Studies have shown that macrophages migrate only through the mechanical traction of the ECM by micro-needles but without the presence of biochemical chemoattractants, confirming that dynamic mechanical signals themselves can form chemotaxis (9).

In recent years, our growing understanding of cellular mechanics has provided us with the opportunity to further investigate the transduction mechanisms of acupuncture signals. Quantitative observations by ultrasound elastography show that when rotating the filiform needle at the acupuncture point, the mechanical coupling effect between the needle and the tissue is significantly enhanced, resulting in the displacement amplitude of the local tissue caused by the lifting and thrusting movement of the needle increasing to several times that without rotation (10). Moreover, the tissue deformation induced by the needling technique not only occurs during the process of needle insertion and withdrawal, but also causes the tissue’s elastic retraction during the needle body’s movement. Acupuncture, in essence, constitutes a highly dynamic mechanical stimulation, wherein the signals propagate through the fibrous network within connective tissue in the form of “vibration waves,” achieving a transmission velocity up to three times that of neural conduction (4). This phenomenon provides theoretical support for explaining the rapid effects of acupuncture: mechanical waves can be rapidly transmitted to distant sites, and through the interaction between the matrix and cells, cells sensitive to mechanical forces release specific biochemical molecules at their sites, further activating other types of cells in the surrounding area, forming a cascade response of the cell population in the acupoint area to acupuncture.

## 3 Fibroblast-mediated mechanical signal transduction

### 3.1 Mechanical sensing and morphological responses of fibroblasts

Fibroblasts not only serve as the primary cells responsible for secreting the ECM, but also adhere to the ECM, enabling them to directly receive and be activated by mechanical signals from acupuncture (11). As such, they constitute the preferential responsive cells to mechanical stimulation induced by acupuncture (5). *In vitro* cellular studies have demonstrated that mechanical stretch stimulation can induce rapid and significant morphological responses in fibroblasts (5). Animal experiments have also demonstrated that the mechanical forces generated by acupuncture significantly alter the morphology and arrangement of fibroblasts in the acupoint region, resulting in spindle-shaped cytoskeletons with denser alignment, increased cross-sectional area, and interconnected cytoskeletal structures with adjacent cells, forming filamentous or reticular networks (12). *In vitro* experiments have shown that a 25% stretching (lasting from 10 minutes to 2 hours) can cause fibroblasts to increase their cell body perimeter and cross-sectional area in a time-dependent manner (13). More convincingly, within seconds to minutes after the acupuncture manipulation, fibroblasts actively respond to tissue stretching deformation through dynamic cytoskeleton remodeling. This rapid cytoskeleton remodeling enables the cross-sectional area of fibroblasts within the tissue plane to expand several times, demonstrating their high mechanical sensitivity (13). Therefore, fibroblasts convert mechanical stimuli into biological signals through signal transduction, thereby altering the microenvironment of the acupuncture area and influencing the activities of other cells, playing a crucial role in the initiation of the acupuncture effect.

### 3.2 Paracrine signaling and immune recruitment by fibroblasts

Fibroblasts activated by mechanical signals transmitted through the ECM are capable of secreting various chemokines and pro-inflammatory factors, thereby recruiting immune cells such as macrophages to cluster in the lesion area, laying the foundation for subsequent immune regulation. For instance, following mechanical stimulation via acupuncture, fibroblasts may release cytokines such as prostaglandin E2 (PGE2), C-C motif chemokine ligand 2 (CCL2), interleukin-6 (IL-6), tumor necrosis factor-alpha (TNF-α), and interferon-gamma (IFN-γ) (14), which attract immune cells, including macrophages, thereby participating in tissue repair and immune responses. In addition to those chemokines, IL-33 is a cytokine that dynamically distributes between the nucleus and cytoplasm of cells and can be secreted by living cells through a non-classical pathway under mechanical stimulation. *In vitro* experiments have shown that the level of extracellular IL-33 significantly increases in skin fibroblasts after periodic biaxial stretching stimulation (15). As suppression of tumorigenicity 2 (ST2) serves as the receptor for IL-33, the transduction of IL-33 signaling is contingent upon the expression of ST2 (16). Transwell migration assays have demonstrated that IL-33 exerts a dose-dependent chemotactic effect on RAW264.7 cells through the expression of ST2 (17), suggesting that macrophage recruitment may depend on the fibroblasts’ IL-33 release during acupuncture. Furthermore, fibroblast contraction generates mechanical forces, which also propagate through the fibrous ECM, providing long-range physical signals to macrophages and directing their migration toward the force source (9).

In summary, current *in vitro* models have provided valuable clues for the explanation of the acupuncture initiation mechanisms, but the *in vivo* evidence that can directly verify these mechanisms is still missing. Moreover, the specific mechanisms by which fibroblasts affect macrophage function are mostly hypothetical at present and urgently need to be confirmed through further studies.

## 4 Mechanical sensing and functional regulation of macrophages

Macrophages, as a type of immune cell sensitive to mechanical signals, perceive mechanical stimuli through their adhesion to the ECM and adjust their migratory behavior accordingly (18). *In vitro* studies have shown that in three-dimensional (3D) cultures, both the fiber structure and the ECM stiffness can regulate the migratory patterns of macrophages: in dense Matrigel or collagen gels, macrophages migrate in an amoeboid pattern, while in loose fibrous collagen gels with larger fiber gaps, they migrate in an interstitial cell pattern. Moreover, individual macrophages can dynamically switch between the two migration modes in response to changes in the matrix architecture (19, 20). In addition, matrix stiffness also affects the cells’ ability to modulate the matrix architecture (such as pore size and rigidity), thus indirectly altering the cell migratory patterns. Interestingly, this phenomenon is not an isolated event; for example, CD4^+^ T cells also follow similar migration rules (21). Therefore, the rheological properties of the ECM play a crucial role in the migration of immune cells.

As an exogenous mechanical intervention, the core physical effect of acupuncture lies in the instantaneous and sustained remodeling of the physical and chemical microenvironment of the ECM through the mechanical coupling between the needle and the tissue. This remodeled mechanical microenvironment can be perceived by highly mechanosensitive macrophages in the tissue through multiple mechanisms in a coordinated manner (22, 23), including integrin-mediated, cytoskeleton-mediated, and podosome-mediated mechanical signal transduction, as well as the rapid activation of mechanically sensitive ion channels such as Piezo1/TRPV4. By converting physical signals into intracellular biochemical signals and integrating them through complex signaling networks, it ultimately drives the process of M1/M2 phenotypic polarization, directional migration, changes in phagocytic activity, and tissue repair function of macrophages, thus playing a core role in the initiation, amplification, and maintenance of the acupuncture effect.

### 4.1 Mechanical sensing pathways of macrophages during acupuncture

#### 4.1.1 Integrin-mediated adhesion and mechanotransduction

**Figure 2:**
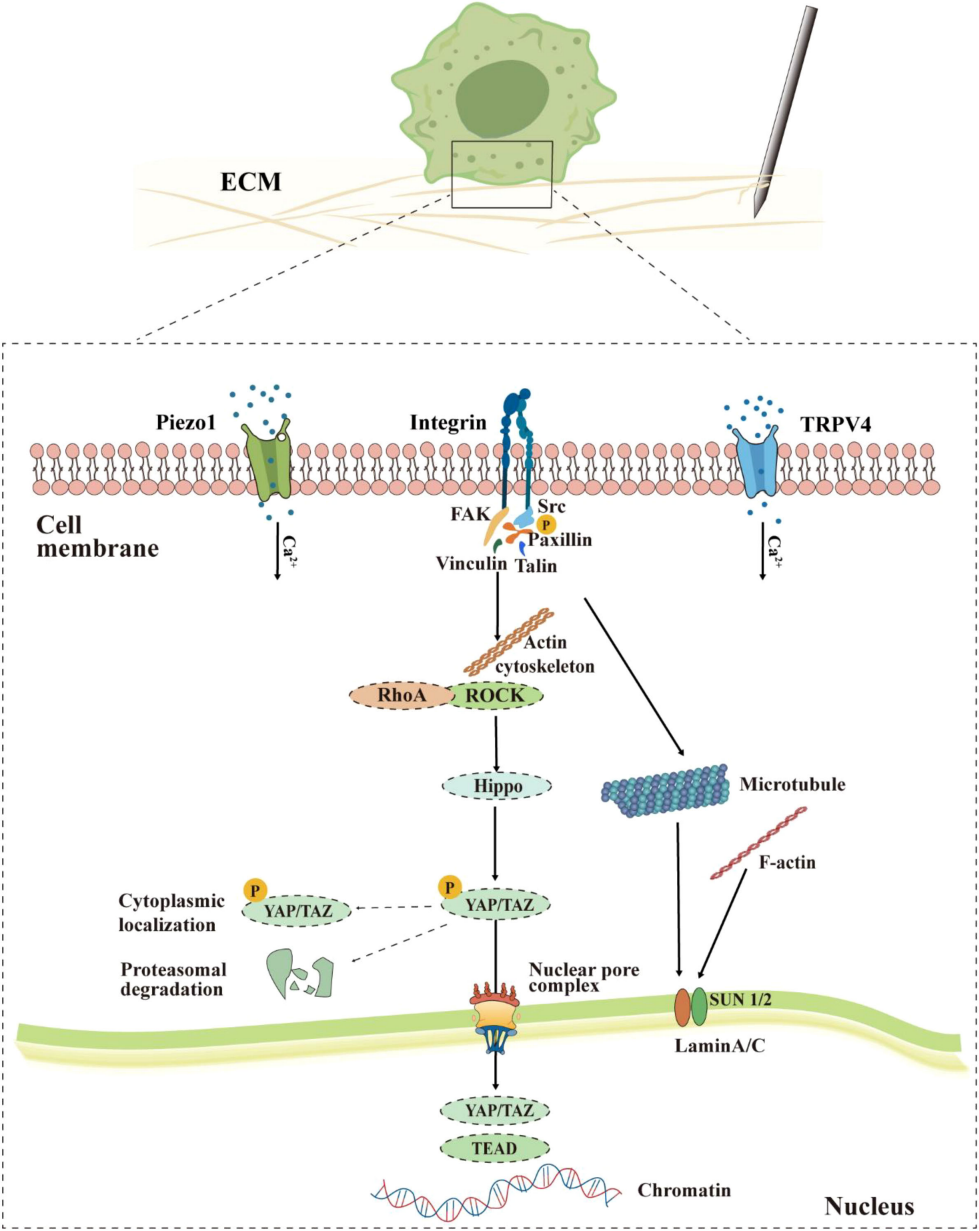
Proposed model of macrophage mechanotransduction during acupuncture. Mechanical stimulation is perceived through integrins, Piezo1/TRPV4 channels, activating downstream signals such as FAK, RhoA/ROCK, and causing dynamic reorganization of the cytoskeleton (actin, microtubules). This process inhibits the retention and degradation of YAP/TAZ in the cytoplasm, promoting their passage through the nuclear pore complex into the nucleus. Within the nucleus, YAP/TAZ interacts with chromatin, while nuclear lamina proteins such as Lamin A/C respond to mechanical signals, jointly mediating the functional reprogramming of macrophages.

While acupuncture produces mechanical stimuli in the acupoint area, the integrins on the macrophages’ cell membrane may be activated and trigger the “ECM-integrin-cytoskeleton-nucleus” mechanotransduction cascade (24) (**Figure 2**). In this process, integrins aggregate and activate in response to the increase in external force. The activated integrins can recruit and activate focal adhesion kinase (FAK) and Src family kinases, forming a FAK-Src signaling complex. This complex then phosphorylates the downstream adaptor protein Paxillin, providing anchor sites for the recruitment of structural proteins such as Vinculin and Talin, ultimately promoting the maturation of focal adhesions and enhancing their roles as a stable anchorage site of the actin cytoskeleton (25). While the Ras homolog family member A (RhoA) is activated through mitogen, its downstream protein Rho−associated protein kinase (ROCK) drives myosin to generate a strong contractile force along the actin cytoskeletal network, directly exerting mechanical traction on the nucleus. On one hand, this contractile force inhibits the Hippo pathway, preventing the degradation of the transcriptional coactivators YAP/TAZ. On the other hand, it promotes the entry of YAP/TAZ into the nucleus, which initiates transcriptional reprogramming and ultimately alters the function of macrophages.

Integrins are transmembrane heterodimeric receptors composed of α and β subunits, serving as the main connectors between the ECM and the cytoskeleton, and playing a crucial role in cell-ECM adhesion and mechanical signal transduction (26). Macrophages may precisely sense the mechanical stimuli from the ECM through adhesion molecules such as integrin (27), converting external mechanical signals into internal biochemical signals, thereby precisely regulating the functions of macrophages, such as adhesion, migration, phagocytosis, and inflammatory responses. Integrin-mediated adhesion can promote the formation of foot processes in macrophages. These structures are not only essential for their migration but are also regarded as potential mechanical sensing units due to their tight connection with the cytoskeleton (28–30).

At the functional level, integrin-mediated adhesion promotes the reorganization and dynamic adjustment of the actin cytoskeleton, which is the structural basis for the mechanical sensing and response of macrophages (27). For instance, the expression levels of β2 and β3 integrins are directly associated with the adhesion and migration capabilities of macrophages, and the inhibition of these integrins can significantly weaken these functions (31–33). In the regulation of phagocytosis, blocking αvβ3 integrin completely inhibits the phagocytosis of fibronectin-coated particles (34). Additionally, integrin signaling possesses a negative regulatory effect on excessive inflammatory activation of macrophages, whereas its absence enhances the production of TNF-α under inflammatory stimulation (35).

Integrin-sensed mechanical signals drive actin cytoskeleton reorganization and enhance cell contractility by activating the RhoA/ROCK pathway. The resulting mechanical tension inhibits the activity of the Hippo pathway downstream kinases LATS1/2, blocking the phosphorylation and cytoplasmic retention of YAP/TAZ. YAP/TAZ undergo nuclear translocation and bind to transcription factors such as TEAD to activate the transcription of target genes that regulate cell growth and polarization (36–39) (**Figure 2**). YAP and TAZ act as key effectors of the Hippo pathway and play a crucial role in the intranuclear transduction of mechanical and cytoskeletal signals (36–38). Their cellular localization is strictly regulated by the mechanical properties of the cell microenvironment. Under conditions of high tension, large cell spreading area, or hard matrix, YAP/TAZ enter the nucleus and activate transcription; while under conditions of soft matrix, small cell area, or high cell density, YAP/TAZ are phosphorylated and retained in the cytoplasm or degraded (36). Twelve hours of mechanical stretching can significantly increase the expression levels of YAP (by approximately 3.2 times) and its upstream regulatory protein Wnt5a (by 2.3 times) in macrophages and promote YAP’s nuclear translocation. Additionally, cyclic mechanical stretching loads can further induce YAP nuclear localization. These evidences support that YAP plays a certain role while macrophages suffer mechanical stimuli (40). During acupuncture, Lamin A/C in macrophages may also sense mechanical signals from the ECM that are transmitted through the integrin-mediated actin-cytoskeleton. Lamin A/C is the main structural protein of the nuclear lamina and serves as an important mechanical sensor within the nucleus. It accurately conveys external mechanical information to the interior of the nucleus, regulates chromatin structure and gene expression, and thus plays an irreplaceable role in maintaining cell morphology, migration, differentiation, and mechanical adaptability (41, 42). Changes in the expression of Lamin A/C can also directly affect the physiological functions of macrophages. Under compressive force stimulation, the levels of SUN1/SUN2 proteins in cells decrease, accompanied by a transient downregulation of Lamin A/C. This downregulation enhances the nuclear localization of yes-associated protein 1 (YAP1), thereby amplifying the effects of mechanical force-induced inflammation and proliferation inhibition (43). Under LPS stimulation, the mRNA and protein levels of Lamin A/C in macrophages show reversible downregulation, accompanied by upregulation of pro-inflammatory gene expression and increased cytokine secretion (44). Inhibition of the activities of cyclin-dependent kinase 1 (CDK1) and Caspase-6 can reduce the production of pro-inflammatory factors IL-6 and TNF-α; while blocking the degradation of Lamin A/C can alleviate the pro-inflammatory response, suggesting its significant role in inflammation regulation (44). Therefore, Lamin A/C constitutes an important bridge connecting extracellular mechanical stimuli with pro-inflammatory gene expression within the nucleus, and its dynamic changes may be the core molecular switch determining whether macrophages maintain a steady state or enter a pro-inflammatory state.

#### 4.1.2 Cytoskeleton-dependent non-classical mechanical sensing mechanism

The cytoskeleton is a highly organized filamentous network within the cells, playing a core role in cell morphology, migration, division, and material transport, and other fundamental life activities. In eukaryotes, the cytoskeleton is composed of three types of cytoskeletal polymers, namely actin filaments, intermediate filaments, and microtubules, as well as three motor protein families, namely myosin, kinesin, and dynein (45, 46). In macrophages, the dynamic reorganization of the cytoskeleton directly participates in regulating their polarized phenotype, ECM degradation, directional migration, and phagocytic activity. Meanwhile, a series of immune responses serves as the chemical stimuli for achieving macrophage recognition, chemotaxis, targeted recruitment, and functional execution (47).

Recent studies have further revealed that macrophages can directly respond to the mechanical properties of the ECM through a non-classical, integrin-independent mechanical sensing mechanism. This process is mediated by the dynamic remodeling of the cytoskeleton during migration, whereas the migration pattern is similar to that of amoeboid movement, which is referred to as “amoeboid-like mechanical sensing” (48). This mechanism involves the direct regulation of the expression of key tissue repair genes, including Fizz1, and does not require the traditional focal adhesion-integrin pathway. In addition, the dynamic changes of the cytoskeleton can also integrate biochemical signals from the microenvironment. For instance, colony-stimulating factor 1 (CSF1) released by activated fibroblasts can induce cytoskeletal remodeling similar to that in high-stiffness ECM and ultimately precisely control the expression program of mechanically sensitive genes by regulating chromatin accessibility, achieving the synergistic integration of mechanical and biochemical signals at the transcriptional level (48).

During acupuncture, the mechanical force generated by the movement of the needle can directly regulate the function of macrophages through the cytoskeleton by altering the physical properties of the local ECM. Meanwhile, CSF1 released by activated fibroblasts can also act on macrophages, and the two work together to initiate and adjust the tissue repair process in response to acupuncture.

#### 4.1.3 Rapid responses mediated by mechanosensitive channels

Piezo1, a calcium-permeable mechanosensitive channel highly expressed in macrophages, serves as a “molecular bridge” coupling physical stimuli with immune responses. *In vitro* experiments have confirmed that the mRNA expression of Piezo1 in the post-acupoint area is significantly upregulated after acupuncture (49). This channel can rapidly open under the action of tissue stretching, shearing, or pressure changes caused by acupuncture, mediating the influx of extracellular Ca²^+^ and initiating calcium-dependent signal cascades at the millisecond level (50). The Piezo1-mediated mechanosensory pathway, along with integrin-dependent signaling, is incorporated into the mechanotransduction model (**Figure 2**), thereby regulating macrophage polarization, phagocytosis, and migration, as well as the secretion of cytokine profiles (51). Additionally, Piezo1 is highly expressed in bone marrow-derived macrophages and participates in regulating multiple functions, including Toll-like receptor 4 (TLR4) signaling, phagocytosis, and bactericidal activity (51). The activation of Piezo1 can promote the activation of calcium-dependent protease Calpain and protein kinase C (PKC), thereby enhancing integrin activation and indirectly influencing macrophage functions (52). Matrix stiffening can also upregulate Piezo1 expression, enabling it to synergize with integrins to activate their common downstream mechanical signaling effector YAP. Activated YAP, as a key regulator of macrophage polarization, can promote their polarization towards the M1 phenotype while inhibiting the M2 phenotype, thus modulating the role of macrophages in immune responses (53, 54). Given the above mechanism, it is reasonable to infer that the mechanical force applied by acupuncture may also be perceived by the Piezo1 and other mechanically sensitive channels of the macrophages in the acupoint area, and trigger a series of similar mechanical signal transduction and functional remodeling.

In addition to Piezo1, macrophages also express multiple TRP family channels, including TRPV2, TRPV4, TRPC6, and TRPM7, which play significant roles in macrophage polarization, inflammatory activation, and phagocytic function (55–60). Among these, TRPV4 is recognized as the primary mechanosensitive TRP channel in macrophages (56). TRPV4 responds to mechanical stimuli such as interstitial fluid flow and changes in substrate stiffness (61). Compared with macrophages cultured on softer substrates, those cultured on harder substrates show significantly enhanced phagocytic ability and intracellular calcium influx after LPS stimulation, and this substrate stiffness effect disappears when TRPV4 is inhibited by drugs or its expression is reduced (56). Hence, TRPV4 works in concert with Piezo1 to enhance macrophage sensitivity to complex mechanical environments by modulating membrane potential and calcium signaling (56, 61–65). Current research has found that acupuncture can activate TRPV channels (especially TRPV4, which is highly expressed in acupoints), mediate the release of ATP in the acupoint area, and thereby produce an analgesic effect of acupuncture (61, 63). However, it is still unknown which specific cells are activated. Additionally, Acupoint Catgut Embedding (ACE), as a novel form of acupuncture, can increase intracellular Ca^2+^ concentration and the number of macrophages by activating TRPV4 (62).

Recent studies indicate that fluid shear stress activates Piezo1, triggering an initial calcium signal that subsequently leads to TRPV4 channel opening and sustained Ca^2+^ elevation (66, 67). The synergistic mechanism of Piezo1 and TRPV4 has not yet been directly confirmed in macrophages or *in vivo* models of acupuncture. However, given that both are key mechanosensitive ion channels and are involved in the regulation of mechanically induced calcium signals in various cell types, we speculate that, in the complex mechanical environment imposed by acupuncture, these two channels may jointly participate in the integration and decoding of mechanical signals through functional synergy. Therefore, Piezo1 and TRPV4 may not only independently mediate rapid mechanical perception and calcium responses, but also potentially form a fine and sensitive mechanical perception network through functional coupling, thereby playing a regulatory role in the immune modulation and tissue repair processes of acupuncture.

In summary, this section integrates the current research on macrophage mechanosensing and proposes a comprehensive model of acupuncture mechanical signal transduction (summarized in **Figure 2**). It should be noted that several key mechanisms involved in this model have been partially verified in different research systems: For instances, the “ECM-integrin-cytoskeleton-nucleus” mechanical transduction pathway and downstream processes such as YAP/TAZ nuclear translocation have been widely explored in cell mechanics research; integrin-independent non-classical mechanosensing mechanisms have also been revealed; the mechanical sensitivity of Piezo1/TRPV4 channels in macrophages and their mediation of rapid Ca^2+^ responses have been supported by *in vitro* experimental results. However, whether these synergistic effects reported in other cell types are also present in macrophages remains unclear. Although *in vitro* studies suggest potential associations among these pathways, the spatiotemporal activation of these molecular events and their functional coupling in the context of *in vivo* acupuncture models still lack systematic evaluation. Future research should leverage live cell imaging acquired in specific gene knockout models and other means to dissect these details.

In essence, the inference here is essentially a reasonable hypothesis based on cross-disciplinary integration, aiming to provide a clear and testable research framework and theoretical basis for exploring the mechanisms specific to the acupuncture context.

#### 4.1.4 Mechanical sensing by podosomes

During the process of acupuncture, the mechanical force applied by the needle causes changes in the mechanical properties of the ECM. Macrophages are likely to detect such alterations directly via podosomes, thereby initiating subsequent mechanotransduction processes.

While adherent cells attach to the ECM through integrins, they recruit adhesion-related proteins to form focal adhesions at the cell membrane, which in turn mediate cytoskeletal remodeling and mechanical signal transduction. In macrophages, this process is primarily carried out by specialized structures known as podosomes. Although similar in protein composition to focal adhesions, the macrophage podosomes exhibit distinct structural morphology and dynamic behavior from those in other cell types (28, 68). Podosomes in macrophages not only participate in cell adhesion but also sense the stiffness and topography of 3D matrices, enabling bidirectional mechanical stress transmission between the intracellular and extracellular environments, and possess the ability to degrade the ECM (28, 69–71).

**Figure 3:**
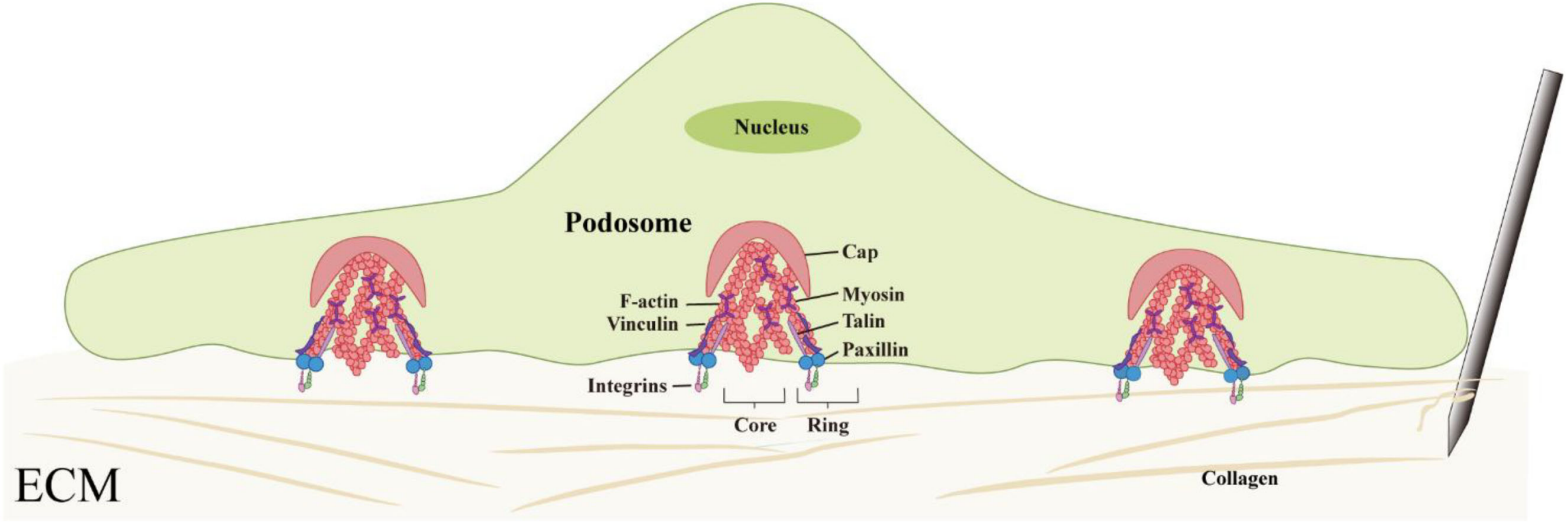
Structural organization and mechanosensory function of macrophage podosomes. Podosomes exhibit a characteristic core–ring–cap architecture composed of actin filaments and adhesion proteins, enabling macrophages to sense matrix stiffness and transmit mechanical forces.

Located at the interface between the cell and the matrix, this structure is a microfilament-rich, spot-like, and with a typical three-layer architecture of “core-ring-cap”: the core is composed of branched actin filaments, surrounded by a ring formed by adhesion proteins (such as talin, Paxillin, and vinculin), and the top is covered by crosslinked and bundled actin filaments to form a cap-like structure. This unique three-dimensional organization provides it with highly sensitive mechanical perception functions (69, 72, 73) (**Figure 3**).

The mechanosensory capacity of podosomes is established through the sustained polymerization of actin filaments combined with myosin-mediated contraction. The detection of activated phosphorylated myosin light chain (pMLC) within and between macrophage podosomes confirms their contractile activity (70). Additionally, in the ring structure of pseudopod bodies, there are mechanically sensitive proteins such as talin and p130Cas that can undergo conformational changes under tension, allowing signal modules such as the binding sites of vinculin or the phosphorylation sites of Src kinase to open after being subjected to force (74, 75). Under the synergistic action of other proteins such as p130Cas, talin, paxillin, and zyxin, mechanical stimuli can be converted into intracellular biochemical signals, completing the transformation from mechanical perception to signal response (70).In summary, the pseudopod body with its characterised “core-ring-cap” ultrastructure, potentially enables macrophages to sensitively detect the mechanical changes in the ECM caused by acupuncture. This structure is believed to not only participate in cell adhesion and migration but also play a role in the perception and transduction of mechanical signals, suggesting that it may be an important structural base for macrophages to achieve mechanosensing (**Figure 3**).

### 4.2 M1/M2 polarization and temporal regulation

As the mechanical stimulation from acupuncture ceases, the biochemical reactions it triggers still undergo a cascade amplification, resulting in a lag effect. Therefore, after acupuncture, the subsequent biochemical reactions still influence macrophage behaviors, including self-chemotaxis, phagocytic activity, antigen presentation, and polarization, laying a functional foundation for regulating other immune cells.

In a mouse model of acute myocardial infarction, after transplanting the infarcted heart into another mouse, the original immune cells (monocytes) in the damaged area of the heart rapidly decreased, while monocytes from the recipient (host) were recruited at an unexpectedly fast rate: 6 hours after transplantation, recipient-derived monocytes accounted for 20 ± 1% of the monocytes in the infarcted area; this proportion increased to 40 ± 3% at 12 hours; and reached 60 ± 0.5% after 24 hours. Hence, the newly recruited monocytes quickly showed a transient residence characteristic in the infarcted myocardial tissue (about 20 hours), maintaining the homeostasis of the local monocyte/macrophage pool in the injury area through this rapid turnover pattern (76). During acupuncture, the immune cells initially recruited by mechanical signals may follow the same rule, and their subsequent M1/M2 phenotypic polarization is a precise functional adjustment and adaptation to the microenvironment of the acupoint area based on this type of dynamic turnover.

Acupuncture may also promote inflammation clearance and tissue repair by regulating the M1/M2 polarization phenotype of macrophages, providing a “function-oriented” guidance for the regulation of other immune cells. Existing research shows that mechanical stress has a bidirectional or even multi-directional effect on macrophage polarization, which is related to the magnitude of the force and other specific factors in the environment. For instance, 12 hours after acupuncture, the phenotype of macrophages changed: in the sepsis model treated with acupuncture, small intestinal macrophages were observed to shift from the pro-inflammatory M1 phenotype to the anti-inflammatory/repair M2 phenotype through immunofluorescence 12 hours after treatment (77). In addition, acupuncture alleviates pain and inflammatory responses by promoting the M1/M2 phenotypic conversion of macrophages and increasing the concentration of IL-10 in muscles (78).

In terms of temporal sequence, the response of skin macrophages to mechanical stress has distinct and dynamic characteristics. Under normal skin conditions, the number of M2-type macrophagesis higher than that of M1-type macrophages (79). However, tissue-resident macrophages release chemokines shortly after being stimulated by acupuncture and then adapt to the changes in the acupoint environment through phenotypic changes over a long period of time. Meanwhile, in the early stage, fibroblasts release pro-inflammatory factors such as IL-6 in response to mechanical stimulation (14). Acupuncture, as a sterile inflammatory operation, damages-associated molecular patterns (DAMPs) released during the acupuncture procedure induce M1 polarization in both tissue-resident macrophages and newly recruited monocytes (80, 81). This M1 polarization leads to the production of mediators such as IL-1β, IL-6, TNF-α, and reactive oxygen species (ROS), nitric oxide (NO), which contribute to tissue repair and regeneration by effectively clearing pathogens and necrotic tissue (82, 83). As the repair process progresses, the concentration of PGE2 in the acupoint area increases (mainly due to the release of fibroblasts under the stimulation of acupuncture), which in turn drives the transformation of macrophages and monocytes into M2 type (14). These transformed cells promote ECM reconstruction, angiogenesis, and tissue regeneration by secreting factors such as TGF-β, IL-10, and VEGF-A (82–84). The phenotypic conversion of macrophages is precisely regulated by microenvironmental signals, but the specific molecular mechanisms and tissue-specific subtype differentiation pathways still need further research.

## 5 Macrophages’ integrated regulation of the acupoint immune microenvironment

After perceiving the mechanical signals of acupuncture, macrophages engaged in adjusting the microenvironment of the acupoint area by altering the chemokine network, leading to the remodeling of the ECM, and transforming other immune cells, thereby transforming the initial mechanical stimulation into a highly regulated immune repair process.

### 5.1 Synergistic regulation of immune cell recruitment and tissue microenvironment remodeling

**Figure 4:**
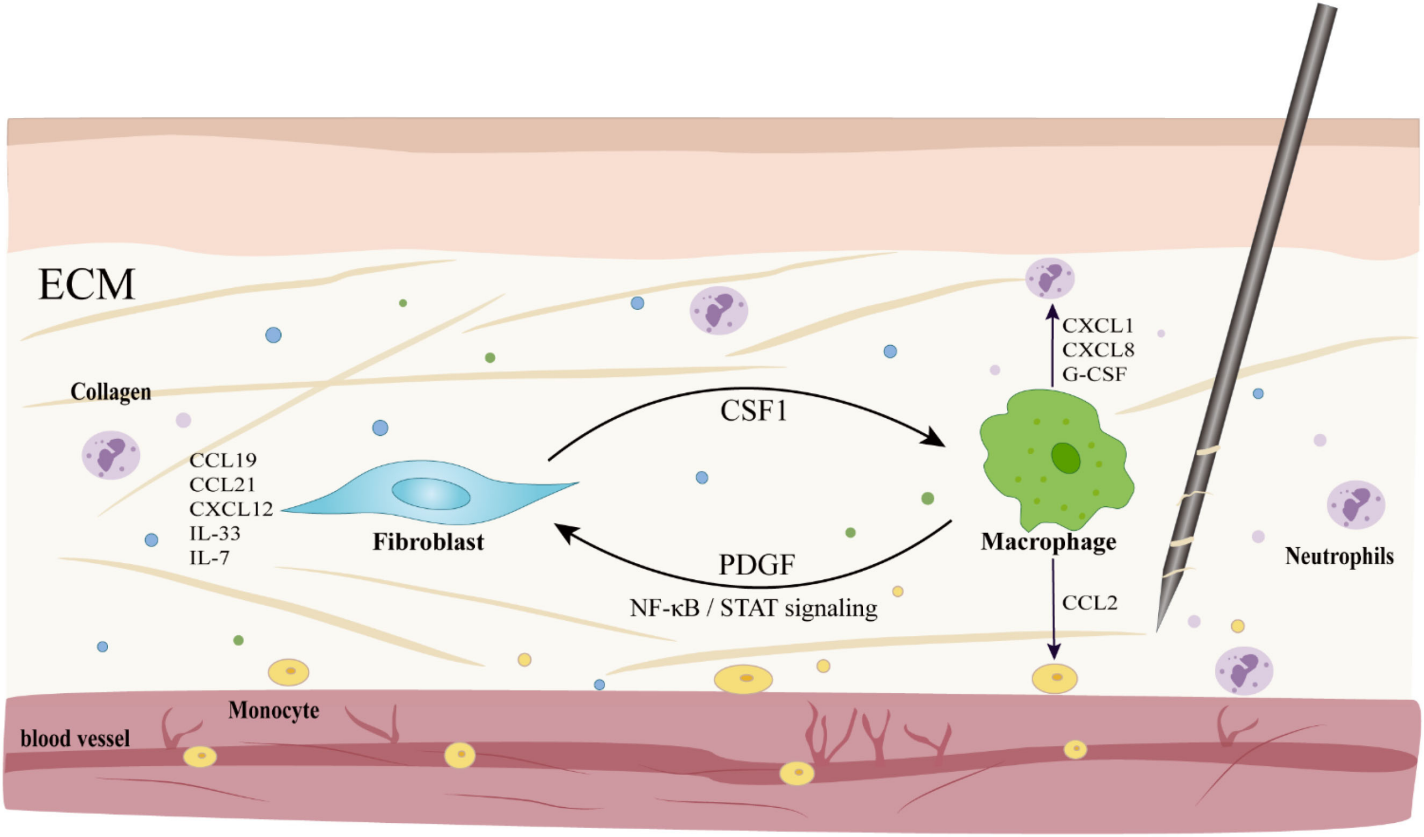
Macrophage-coordinated immune cell recruitment and intercellular communication in the microenvironment of acupoints. Activated macrophages release chemokines such as CXCL1 and CCL2 to recruit neutrophils and monocytes, respectively; meanwhile, fibroblasts secrete factors like IL-33 to exert chemotactic effects and act as “location markers”. The two form a positive feedback through molecules such as CSF1-PDGF, and under the regulation of signaling pathways such as NF-κB/STAT, they jointly maintain the immune homeostasis of the local microenvironment.

In the chemokine network, macrophages act as rapid response units, releasing chemokines such as CXCL1 and CCL2 upon activation of the Piezo1 channel, which respectively recruit neutrophils, monocytes and T cells, initiating the early immune response; while fibroblasts serve as “location markers”, secreting cytokines such as CCL19, CCL21, CXCL12, IL-33 and IL-7 under steady state conditions, maintaining the positioning and functional continuity of immune cells (15, 85–88). Both of them form a positive feedback loop through factors such as CSF1-PDGF and, under the regulation of signaling pathways such as NF-κB and STAT, coordinate the orderly transition of immune cells from recruitment to repair (85, 86). Macrophages rapidly recruit monocytes from the bone marrow and spleen by releasing chemokines such as CCL2 (89), and these monocytes differentiate into macrophages or dendritic cells in the lesion area (90), promptly replenishing the local immune cell count and performing functions such as phagocytosis, antigen presentation, inflammatory regulation, and tissue repair (90–93). In addition, macrophages also enhance the immune defense capacity of the acupoint area by secreting cytokines such as CXCL8 and G-CSF, in collaboration with neutrophils (94, 95) (**Figure 4**). During the ECM remodeling process, fibroblasts respond to mechanical and inflammatory signals, synthesize and deposit ECM components such as collagen (87, 88), and release prostaglandin E2 (PGE2) to promote tissue repair and angiogenesis (14). Macrophages promote the migration of immune cells to the injury site by decomposing the ECM, clearing pathogens, apoptotic cells, and ECM degradation products, and provide a stable microenvironment for tissue repair (95). These two cells work in concert to reconstruct the ECM (87). On one hand, macrophages secrete TGF-β1 to induce fibroblasts-to-myofibroblasts transformation, promoting collagen deposition and tissue repair (82, 96); while the M1/M2 polarization phenotypes cooperatively promote angiogenesis and ECM reconstruction through differentially secreting angiogenic factors and matrix remodeling proteins, such as matrix metalloproteinase-9 (MMP-9) (97). On the other hand, fibroblasts maintain the homeostasis of ECM metabolism by regulating the balance of matrix metalloproteinases (MMPs), tissue inhibitors of MMPs (TIMPs) (88). During the resolution phase of inflammation, macrophages promote the repair process by phagocytosing apoptotic neutrophils and regulating IL-10 and IL-12 (98, 99). If this coordinated mechanism is imbalanced, it can lead to abnormal deposition or degradation of the ECM, thereby inducing pathological outcomes such as fibrosis.

In coordinating innate immune cells, as the main scavenger of senescent and apoptotic neutrophils, macrophages acquire powerful neutrophilic antibacterial molecules, thereby enhancing their own antibacterial capacity (100). The transformation of local macrophages from the early inflammatory M1 phenotype to the later repetitive M2 phenotype at the injury site depends on their phagocytic effect on apoptotic neutrophils. Apoptotic neutrophils release “eat-me” signals, which induce macrophages to upregulate the expression of phagocytosis-related genes and release repair-related cytokines, such as IL-10 and TGF-β (99). The efficient collaboration between macrophages and neutrophils significantly accelerates the resolution of inflammation and the restoration of tissue homeostasis, laying a solid foundation for the sustained effect of acupuncture.

Macrophages act as rapid response units, initiating the recruitment of immune cells by releasing chemokines such as CXCL1 and CCL2. They form a synergy with fibroblasts, which serve as “location markers”, through a cytokine network and positive feedback loop, jointly regulating the progression from inflammation to repair. The interaction between the two in cell communication and ECM remodeling constitutes the potential cellular basis of the acupuncture effect. However, the current understanding mainly stems from *in vitro* experiments and extended inferences from other disease models. The dynamic response patterns, interaction details, and exact contributions of these two types of cells under acupuncture-specific stimulation, as well as their roles in the overall effect, still require rigorous *in vivo* acupuncture research for empirical validation.

### 5.2 Regulation of adaptive immunity

Macrophages are not only the core effector cells of innate immunity but also the key hub connecting innate and adaptive immunity. They mainly regulate T-cell responses precisely through antigen presentation and cytokine secretion, thereby guiding the acupuncture-induced local inflammation towards immune homeostasis and tissue repair.

**Figure 5:**
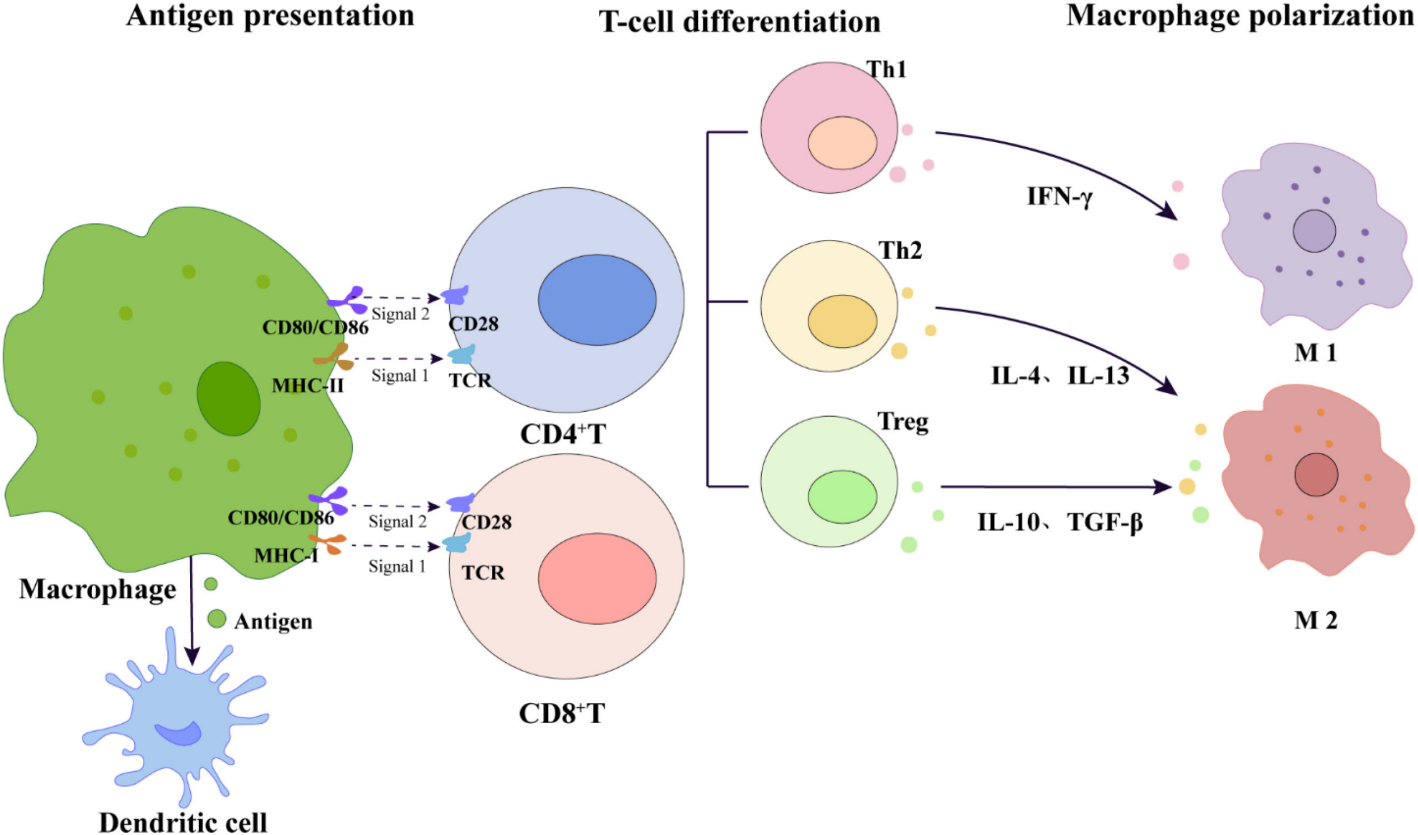
Macrophage-mediated T-cell dual-signal activation and subsequent cytokine-driven differentiation and macrophage polarization. This schematic diagram illustrates the dual-signal activation model of T cells: Signal 1 is the presentation of antigen peptides to the T cell receptor (TCR) by MHC class I and MHC class II molecules on the surface of macrophages; Signal 2 is transmitted through the co-stimulatory interaction between CD80/CD86 on the surface of macrophages and CD28 on the surface of T cells. Activated CD4^+^T cells further differentiate into different effector subgroups, including Th1 (secreting IFN-γ), Th2 (secreting IL-4 and IL-13), and Treg (secreting IL-10 and TGF-β), which feed back to regulate the polarization of macrophages to M1 or M2 phenotypes, thereby maintaining overall immune homeostasis. The diagram also indicates the pathway by which macrophages transfer antigens to dendritic cells to support cross-presentation, jointly participating in the homeostatic regulation after immune activation induced by acupuncture.

In terms of antigen presentation, macrophages are important antigen-presenting cells (APCs) (101), presenting antigen peptides via MHC class I and MHC class II molecules on their surface, providing the essential “first signal” for T cell activation (101, 102). The T cell receptor (TCR) on the T cell membrane recognizes the pMHC complex on the surface of APCs, with CD4+ T cells mainly recognizing antigens presented by MHC class II molecules, while CD8+ T cells mainly recognize antigens presented by MHC class I molecules (101–103). Beyond TCR binding to pMHC, full T cell activation also requires co-stimulatory signals (the “second signal”). This signal is typically provided by the binding of co-stimulatory molecules on APCs, such as CD80/CD86, to the CD28 receptor on T cells (101–103). Under the combined action of both signals, naive T cells become activated and can further differentiate into distinct effector subsets with specific functions, such as helper T cells (including Th1 and Th2) or regulatory T cells (Treg), under the regulation of specific cytokine environments (104) (**Figure 5**). Activated Th1 cells secrete large amounts of IFN-γ, further promoting macrophage polarization toward the pro-inflammatory M1 phenotype and enhancing their phagocytic and bactericidal capabilities. Conversely, Th2 cells further promote the polarization of macrophages to the repair-oriented M2 phenotype by secreting factors such as IL-4 and IL-13, participating in tissue repair and other processes. Meanwhile, Treg cells effectively suppress excessive inflammatory responses by secreting inhibitory cytokines such as IL-10 and TGF-β, maintaining immune homeostasis and self-tolerance in the body (105–107). Additionally, macrophages can also indirectly enhance the efficiency and specificity of antigen presentation by transferring pre-processed antigen fragments to dendritic cells, promoting cross-presentation and preventing the spread of immune responses to non-target tissues (108).

Macrophages activate T cells through antigen presentation and co-stimulatory signals, while activated T cells feed back to regulate the polarization state of macrophages by secreting specific cytokines, forming a bidirectional regulatory network (**Figure 5**). This finely interlocked mechanism is of great significance for translating the local immune response triggered by acupuncture into microenvironmental repair. However, the spatiotemporal dynamic changes of this network under acupuncture stimulation, the sequence of key molecular events, and their specific pathways of action in the overall effect still need to be systematically clarified through the construction and utilization of *in vivo* research models specific to acupuncture.

## 6 Summary and prospect

This review systematically demonstrates that there exists a complete mechanical-biological signal transduction pathway for the initiation of acupuncture. This pathway is initiated by the mechanical force generated through the manipulation of filiform needles, which alters the mechanical microenvironment of the ECM. Macrophages and fibroblasts act as core sensors and respond in a coordinated manner, thereby completing the transduction and amplification from physical stimulation to biochemical signals. Macrophages, as the core hub, sense stress signals through various pathways such as Piezo1/TRPV4 mechanically sensitive channels, integrins, and podosomes, and promptly initiate a cascade of intracellular Ca^2+^ fluctuations and YAP/TAZ nuclear translocation responses. Furthermore, they recruit neutrophils and monocytes by secreting chemokines (such as CCL2, CXCL1, *etc*), initiating innate immunity. Their function then shifts from rapid recruitment to fine regulation. By means of the dynamic polarization of M1/M2 phenotypes and the secretion of factors such as TGF-β and IL-10, it not only guides the differentiation of monocytes into repair-type macrophages but also bridges and activates T cells through antigen presentation, thus establishing a complete “innate-adaptive immune” regulatory network. Meanwhile, macrophages and fibroblasts collaborate precisely in the remodeling of the ECM. The former mediates degradation and clearance, whereas the latter governs synthesis and deposition. Together, they optimize the mechanical microenvironment of the lacunae, driving local immune homeostasis and tissue repair.

Looking ahead, research on mechanical signal transduction and immune regulation in acupuncture still faces several key challenges. Current studies mostly employ single or static mechanical stimulation models, which are unable to reflect the dynamic interaction of multiple parameters, such as intensity, frequency, duration, and technique during clinical acupuncture, as well as their overall regulation of macrophage function and the immune network. Moreover, there is a significant gap between the parameter settings of existing *in vitro* mechanical models and actual acupuncture operations, which hinders the in-depth explanation of related mechanisms and their clinical translation potential.

The realization of acupuncture effects relies on the coordinated actions of various cells in the acupoint area, but the specific mechanisms are not yet fully understood. For instance, how mechanically activated fibroblasts regulate the recruitment and polarization of macrophages through chemokine signals such as IL-33; what molecular dialogues between fibroblasts and macrophages coordinate the regulation of ECM remodeling and the immune microenvironment; how macrophages activate T cells through antigen presentation, and how activated T cells, in turn, regulate macrophage function through cytokine feedback to jointly maintain immune homeostasis. These key links urgently need to be systematically verified through acupuncture-specific *in vivo* models. At the same time, the temporal activation sequence, cooperative mechanisms, and their impact on the final immune outcome of multiple mechanical sensing pathways, such as Piezo1/TRPV4 channels and integrin-cytoskeleton-nuclear signaling axes during the acupuncture process, also need to be further systematically clarified.

Therefore, future research should establish a standardized mechanical stimulation system for acupuncture that is closer to clinical reality, and integrate *in vivo* models with live imaging techniques to analyze the dynamic impact of mechanical input on immune responses from a spatiotemporal perspective. The focus should be on building acupuncture-specific *in vivo* research systems, integrating cell-specific intervention and real-time dynamic observation techniques, to systematically reveal the integration mechanism of mechanical signals in the microenvironment of the acupoint area and their precise regulatory rules on immune cell behavior and tissue repair programs, thereby providing a solid scientific basis for the modernization and precision of acupuncture therapy.
